# A non-field analytical method for heat transfer problems through a moving boundary

**DOI:** 10.1038/s41598-021-98572-x

**Published:** 2021-09-23

**Authors:** Vladimir Kulish, Vladimír Horák

**Affiliations:** 1 Department of Thermodynamics and Fluid Mechanics, Faculty of Mechanical Engineering, Czech Technical University, Prague, Czech Republic; 2 Department of Mechanical Engineering, Faculty of Military Technology, University of Defence, Brno, Czech Republic

**Keywords:** Mechanical engineering, Thermodynamics

## Abstract

This paper presents an extension of the non-field analytical method—known as the method of Kulish—to solving heat transfer problems in domains with a moving boundary. This is an important type of problems with various applications in different areas of science. Among these are heat transfer due to chemical reactions, ignition and explosions, combustion, and many others. The general form of the non-field solution has been obtained for the case of an arbitrarily moving boundary. After that some particular cases of the solution are considered. Among them are such cases as the boundary speed changing linearly, parabolically, exponentially, and polynomially. Whenever possible, the solutions thus obtained have been compared with known solutions. The final part of the paper is devoted to determination of the front propagation law in Stefan-type problems at large times. Asymptotic solutions have been found for several important cases of the front propagation.

## Introduction

Heat transfer in domains with a moving boundary is an important problem with various applications in different areas of science. Among these are, for instance, heat transfer due to chemical reactions, ignition and explosions, combustion, and many others.

It is known since long that finding closed-form solutions of the heat equation with the convective term is an extremely challenging task^1^. In some cases, these analytical solutions can be found by the use of thermal potentials, but, even then, such an approach, in general, results into a set of integral equations rather than a single equation and worse still, requires additional auxiliary unknown functions (densities of the thermal potentials of the single and double layers) to be introduced^2,3^.

In most of the classical texts, theoretical investigations of the Stefan problem are focused on the situation, where the boundary moves with a constant speed^4,5^. The same situation exists in recent studies of advanced forms of the Stefan problem^6–9^. Even in the most recent study, in which the phase-change temperature is size or velocity dependent, the assumption of the boundary speed constancy has been made^10^. Thus, no theoretical investigations of the Stefan problem are known, in which the boundary speed would be arbitrary.

This paper presents an elegant single-formula solution of the heat equation with the convective term, in which the speed varies arbitrarily. This solution is obtained by the method nowadays known as the method of Kulish^11^. The method renders non-field solutions of partial differential equations. These solutions are so called, because they relate local values of temperature and the temperature gradient, and thus there is no need for determining the entire temperature field. Over the past 2 decades, the non-field method has been successfully applied for solving problems in different areas of science ranging from thermal-fluid sciences^12–15^ to biomedical engineering^16^ and even econometrics^17^. In the most recent study, a theoretical justification of the method together with the general expression for the involved fractional operator has been provided^18^.

It has been shown^18^ that the non-field solution of the transport equation (the heat equation in this study)

1 $$ \left[ {\frac{\partial }{\partial t} - \alpha \left( {x,t} \right)\frac{{\partial^{2} }}{{\partial x^{2} }} - \beta \left( {x,t} \right)\frac{\partial }{\partial x} + \gamma \left( {x,t} \right)} \right]T = 0 $$

is given in the form^18^

2 $$ - q\left( {x,t} \right)\sqrt {\alpha \left( {x,t} \right)} = \mathop {\mathop \sum \limits_{n = 0} }\limits^{\infty } a_{n} \left( {x,t} \right)\partial^{{\left( {1 - n} \right)/2}} T\left( {x,t} \right), $$

where ${\partial }^{1-n/2}$ denotes fractional derivatives defined through the Riemann–Liouville integrals as^18^

3 $$ \partial^{\nu } f\left( t \right) = \frac{{d^{\nu } f\left( t \right)}}{{dt^{\nu } }} = \frac{1}{{\Gamma \left( {1 - \nu } \right)}}\frac{d}{dt}\int_{0}^{t} \frac{f\left( \zeta \right)d\zeta }{{(t - \zeta )^{\nu } }},\,\,\,\, - \infty < Re\left( \nu \right) < 1. $$

Notice that not only Eq. (2) is valid within the entire domain but also remains valid on the domain boundary (surface), $x=0$, that is,

4 $$ - q_{s} \left( t \right)\sqrt {\alpha \left( {0,t} \right)} = \mathop {\mathop \sum \limits_{n = 0} }\limits^{\infty } a_{n} \left( {0,t} \right)\partial^{{\frac{1 - n}{2}}} T_{s} \left( t \right) $$

It is worth noting here that the non-field method, discussed in this study, yields the solution of Eq. (1) given in the form of Eq. (2) for any boundary conditions imposed on Eq. (1). Hence, there is no need to specify a set of boundary conditions for the said equation. Moreover, as can be easily seen from Eq. (4), the method can be used to transform the Direchlet boundary conditions into the Neumann ones and vice versa^12,13^.

Equations (2) and (4) can be obtained because the original transport Eq. (1) is reducible to the corresponding fractional partial differential equation

5 $$ \left[ {\frac{\partial }{\partial t} - \sqrt \alpha {\mathfrak{D}}\left( {\frac{1}{\sqrt \alpha }{\mathfrak{D}}} \right) + \sqrt \alpha \left( {\frac{{\partial {\mathfrak{D}}}}{\partial x}} \right) - \frac{1}{2\sqrt \alpha }\frac{\partial \alpha }{{\partial x}}{\mathfrak{D}} + \frac{\beta }{\sqrt \alpha }{\mathfrak{D}} + \gamma } \right]T = 0, $$

where the operator

6 $$ {\mathfrak{D}} = \mathop {\mathop \sum \limits_{n = 0} }\limits^{\infty } a_{n} \left( {x,t} \right)\partial^{{\left( {1 - n} \right)/2}} $$

is given as a series with respect to fractional derivatives.

It has been shown also that the recurrent expressions for the coefficients ${a}_{n}$ in Eqs. (2), (4), and (6) are as follows:

7 $$ \begin{aligned} \partial & :1 - a_{0}^{2} = 0 \\ \partial^{1/2} & :\sqrt \alpha \frac{{\partial a_{0} }}{\partial x} + \frac{\beta }{\sqrt \alpha }a_{0} - \frac{1}{2\sqrt \alpha }\frac{\partial \alpha }{{\partial x}} - a_{1} a_{0} - a_{0} a_{1} = 0 \\ \partial^{0} & :\sqrt \alpha \frac{{\partial a_{1} }}{\partial x} + \frac{\beta }{\sqrt \alpha }a_{1} - \frac{1}{2\sqrt \alpha }\frac{\partial \alpha }{{\partial x}}a_{1} - \gamma - a_{2} a_{0} - a_{1}^{2} - a_{0} a_{2} = 0 \\ \partial^{{\left( {1 - k} \right)/2}} & :\sqrt \alpha \frac{{\partial a_{k} }}{\partial x} + \frac{\beta }{\sqrt \alpha }a_{k} - \frac{1}{2\sqrt \alpha }\frac{\partial \alpha }{{\partial x}}a_{k} \\ & \quad - \sqrt \alpha \mathop {\mathop \sum \limits_{n = 0} }\limits^{n + 2m} \mathop {\mathop \sum \limits_{m = 0} }\limits^{ \le k + 1} \left( {\begin{array}{*{20}c} {\frac{1 - n}{2}} \\ m \\ \end{array} } \right)a_{n} \frac{{\partial^{m} }}{{\partial t^{m} }}\left( {\frac{{a_{k - 1 - n - 2m} }}{\sqrt \alpha }} \right) = 0,\,\,\,\,\,k \ge 2 \\ \end{aligned} $$

In the following sections, it is shown how the above results can be extended to some heat transfer problems in domains with a moving boundary.

The latter case is modelled by the heat equation, in which the coefficients $\alpha ,\beta , {\text{and}} \, \gamma$ depend on time only. In such a case, the fractional operator can be expressed in an alternative form, which enables improved convergence of final results. Section “An alternative representation of fractional operators: improved convergence” discusses this alternative form of the fractional operator.

Section “Heat transfer through a moving boundary” is devoted to establishing the non-field solution of the heat equation, which models the process of heat transfer into a semi-infinite domain through the arbitrarily moving boundary. Some particular cases of the solution are considered in the end of the section. Among them are such cases as the boundary speed changing linearly, parabolically, exponentially, and polynomially.

In section “Determination of the front propagation law in Stefan-type problems at large times”, some Stefan-type problems are considered. Most of this section is devoted to determination of the front propagation law at large times. Asymptotic solutions have been found for several important cases of the front propagation.

## An alternative representation of fractional operators: improved convergence

First, consider the case, when all the coefficients in the original Eq. (1) are constant, that is, $\alpha =const$, $\beta =const$, and $\gamma =const$. In this case, the solution, obtained by the non-field method can be compared with the exact solution found by means of Laplace transforms. Indeed, Eqs. (4) and (7) render

8 $$ \begin{aligned} - q_{s} \sqrt \alpha & = \partial^{1/2} + \frac{\beta }{2\sqrt \alpha } + \frac{\delta }{2}\partial^{ - 1/2} - \frac{{\delta^{2} }}{8}\partial^{ - 3/2} + \cdots \\ & = \frac{1}{{\sqrt {\pi t} }} + \frac{\beta }{2\sqrt \alpha } + \delta \sqrt {\frac{t}{\pi }} - \frac{{4\delta^{2} }}{24\sqrt \pi }t^{3/2} + \cdots ,\,\,\,\,\delta = \gamma + \frac{{\beta^{2} }}{4\alpha } \\ \end{aligned} $$

while the method of Laplace transforms yields

9 $$ \begin{aligned} - \sqrt \alpha \frac{d\Theta }{{dx}}|_{x = 0} & = \frac{1}{p}\left( {\sqrt {p + \delta } + \frac{\beta }{2\sqrt \alpha }} \right) \\ & = \frac{1}{\sqrt p } + \frac{1}{p}\frac{\beta }{2\sqrt \alpha } + \frac{1}{{p^{3/2} }}\frac{\delta }{2} - \frac{1}{{p^{5/2} }}\frac{{\delta^{2} }}{8} + \ldots \\ \end{aligned} $$

where $\Theta ={\mathfrak{L}}_{t}[T]$ denotes the Laplace transform of the temperature.

Notice that, in Eqs. (8) and (9), ${T}_{s}=1$ for the sake of simplicity but without lose of generality.

Taking into account that ${\mathfrak{L}}_{t}[{t}^{\nu }]=\Gamma (\nu +1)/{p}^{\nu +1}$, Eqs. (8) and (9) are identical.

Upon invoking Eq. (3), the series in the right side of Eq. (8) can be summed up, that is,

10 $$ - q_{s} \sqrt \alpha = e^{ - \delta t} \left( {\partial^{1/2} + \frac{\beta }{2\sqrt \alpha }} \right)e^{\delta t} T_{s} \left( t \right) $$

In comparison with Eq. (8), the latter expression is much more convenient, especially for large values of $t$.

The latter result suggests that, in the case of $\alpha =const$, $\beta =const$, and $\gamma =const$, the operator in the fractional representation of the transport equation, Eq. (2), can be written as

11 $$ \left[ {e^{ - \delta t} \left( {\partial^{1/2} + \frac{\beta }{2\sqrt \alpha }} \right)e^{\delta t} + \sqrt \alpha \frac{\partial }{\partial x}} \right]T = 0 $$

For Eq. (2) with the arbitrary coefficients, the corresponding fractional form is

12 $$ \left[ {exp\left( { - \int_{0}^{t} \delta \left( \zeta \right)d\zeta } \right)\mathop {\mathop \sum \limits_{n = 0} }\limits^{\infty } a_{n}^{*} \partial^{{\left( {1 - n} \right)/2}} exp\left( {\int_{0}^{t} \delta \left( \zeta \right)d\zeta } \right) + \sqrt \alpha \frac{\partial }{\partial x}} \right]T = 0, $$

where the coefficients ${a}_{n}^{*}$ differ from those in Eqs. (4) and (6) but can be determined by the same methods as the coefficients ${a}_{n}$. In the following sections, these coefficients are determined separately for each case considered.

As can be easily seen from Eq. (12), the corresponding non-field solution of the original transport Eq. (1) at the surface becomes

13 $$ - q_{s} \sqrt \alpha = \left[ {exp\left( { - \int_{0}^{t} \delta \left( \zeta \right)d\zeta } \right)\mathop {\mathop \sum \limits_{n = 0} }\limits^{\infty } a_{n}^{*} \partial^{{\left( {1 - n} \right)/2}} exp\left( {\int_{0}^{t} \delta \left( \zeta \right)d\zeta } \right)} \right]T_{s} = 0 $$

It is worth noting here that, for practical purposes, convergence of the series in Eqs. (12) and (13) for large values of $t$ is better than in Eq. (6)—in particular, when $\alpha ,\beta ,{\text{and}}\gamma$ are slowly varying functions.

To conclude this section, it is worth noting that the result, presented above, is important from the methodological point of view. This is so, because the alternative form of the fractional operator given by Eq. (12) is established here for the first time. In comparison with the generalisation of the method reported reported earlier^18^, the alternative form warrants improved convergence of the method. The latter is explored in the second part of sections “Heat transfer through a moving boundary” and “Determination of the front propagation law in Stefan-type problems at large times”.

## Heat transfer through a moving boundary

Consider the process of heat transfer into a semi-infinite domain through the boundary, which moves arbitrarily as ${\fancyscript{l}}={x}_{0}-{\int }_{0}^{t}\beta (\zeta )d\zeta$. Then, in the coordinate system related to the boundary, the heat transfer problem becomes identical to Eq. (1) with $\alpha =const$, $\beta =\beta (t)$, $\gamma =0$. The latter problem is of great importance while modelling processes of propagation of the phase transition fronts, chemical reactions, or combustion.

If the coefficients in the original Eq. (1) depend on time only, as is the case for the problem in question, Eq. (5) reduces to

14 $$ \left[ {\frac{\partial }{\partial t} - \sqrt \alpha {\mathfrak{D}}\left( {\frac{1}{\sqrt \alpha }{\mathfrak{D}}} \right) + \frac{\beta }{\sqrt \alpha }{\mathfrak{D}} + \gamma } \right]T = 0. $$

Consequently, the recurrent expressions given by Eq. (7) simplify into

15 $$ \begin{aligned} \partial & :a_{0} = 1 \\ \partial^{1/2} & : - 2a_{1} + \frac{\beta }{\sqrt \alpha } = 0 \\ \partial^{0} & : - 2a_{2} - a_{1}^{2} + \frac{\beta }{\sqrt \alpha }a_{1} + \gamma = 0 \\ \partial^{{\left( {1 - k} \right)/2}} & : - \mathop {\mathop \sum \limits_{n = 0} }\limits^{n + 2m} \mathop {\mathop \sum \limits_{m = 0} }\limits^{ \le k + 1} \left( {\begin{array}{*{20}c} {\frac{1 - n}{2}} \\ m \\ \end{array} } \right)a_{n} \frac{{d^{m} }}{{dt^{m} }}\left( {\frac{{a_{k + 1 - n - 2m} }}{\sqrt \alpha }} \right) + \frac{\beta }{\sqrt \alpha }a_{k} = 0,\,\,\,k \ge 2 \\ \end{aligned} $$

Then, from Eqs. (4) and (15), the expression for the temperature gradient at the surface, ${q}_{s}=(\partial T/\partial x{)}_{x=0}$, is in the form

16 $$ \begin{aligned} - \frac{{q_{s} }}{{T_{s} }}\sqrt \alpha & = \partial^{1/2} + \frac{\beta }{2\sqrt \alpha } + \frac{{\beta^{2} }}{8\alpha }\partial^{ - 1/2} - \frac{\beta }{8\sqrt \alpha }\partial^{ - 1} - \left( {\frac{{\beta^{4} }}{{128\alpha^{2} }} + \frac{{\mathop \beta \limits^{\cdot} \beta }}{16\alpha }} \right)\partial^{ - 3/2} \\ & \quad + \left( {\frac{{\beta^{2} \dot{\beta }}}{{32\alpha^{3/2} }} + \frac{{\ddot{\beta }}}{16\sqrt \alpha }} \right)\partial^{ - 2} + \left( {\frac{{\beta^{6} }}{{1024\alpha^{3} }} + \frac{{3\beta^{3} \dot{\beta }}}{{128\alpha^{2} }} - \frac{{\dot{\beta }^{2} }}{128\alpha } + \frac{{\beta \ddot{\beta }}}{32\alpha }} \right)\partial^{ - 5/2} \\ & \quad - \left( {\frac{{\beta^{4} \dot{\beta }}}{{128\alpha^{5/2} }} + \frac{{\beta \dot{\beta }^{2} }}{{16\alpha^{3/2} }} + \frac{{\beta^{2} \ddot{\beta }}}{{32\alpha^{3/2} }} + \frac{5\dddot \beta }{{128\sqrt \alpha }}} \right)\partial^{ - 3} \\ & \quad - \left( {\frac{{5\beta^{8} }}{{2^{15} \alpha^{4} }} + \frac{{15\beta^{5} \dot{\beta }}}{{2048\alpha^{3} }} + \frac{{\beta^{3} \ddot{\beta }}}{{64\alpha^{2} }} + \frac{{13\beta^{2} \dot{\beta }^{2} }}{{1024\alpha^{2} }} - \frac{{5\dot{\beta }\ddot{\beta }}}{{128\alpha^{2} }} + \frac{5\beta \dddot \beta }{{256\alpha }}} \right)\partial^{ - 7/2} + \left( \cdots \right)\partial^{ - 4} + \cdots \\ \end{aligned} $$

where dots denote derivatives with respect to time.

As an example, consider the case ${T}_{s}=1$ and $\beta =C/\sqrt{t}$ with $C=const$.

Taking into account that fractional derivatives of power functions have a very simple expression, namely^19^:

17 $$ \partial^{\nu } t^{\mu } = \frac{{\Gamma \left( {\mu + 1} \right)}}{{\Gamma \left( {\mu + 1 - \nu } \right)}}t^{\mu - \nu } $$

from (16), it follows that

18 $$ \begin{aligned} - q_{s} \sqrt t & = \frac{1}{\sqrt \pi } + \frac{C}{\sqrt \alpha }\left( {\frac{1}{2} + \frac{1}{2} + \frac{3}{18} + \frac{25}{{2048}} + \frac{245}{{32768}} + \cdots } \right) \\ & \quad + \frac{{C^{2} }}{\alpha \sqrt \pi }\left( {\frac{1}{4} + \frac{1}{24} + \frac{1}{840} + \frac{45}{{2048}} + \cdots } \right) + \cdots \\ \end{aligned} $$

As can be easily seen, in this case, all the terms in series (18) have the same power $1/\sqrt{t}$.

To obtain solutions in more complicated cases, it is much more useful to look for these solutions in the form with improved convergence.

To find the corresponding alternative form of the fractional operator (12) in the case of $\alpha =const$, $\beta =\beta (t)$, $\gamma =0$, substitute

19 $$ {\mathfrak{D}} = exp\left( { - \int_{0}^{t} \frac{{\beta^{2} }}{4}d\zeta } \right){\mathfrak{F}}exp\left( {\int_{0}^{t} \frac{{\beta^{2} }}{4}d\zeta } \right) $$

into Eq. (5).

Transition from the operator $\mathfrak{D}$ to the operator $\mathfrak{F}$ corresponds to the change of variable $\Theta =Texp\left[{\int }_{0}^{t}({\beta }^{2}/4)d\zeta \right]$.

Then, the equation for the operator $\mathfrak{F}$ becomes

20 $$ {\mathfrak{F}}^{2} - \beta \left( t \right){\mathfrak{F}} + \frac{1}{4}\beta^{2} \left( t \right) = \partial , $$

where

21 $$ {\mathfrak{F}} = \mathop {\mathop \sum \limits_{n = 0} }\limits^{\infty } a_{n}^{*} \left( t \right)\partial^{{\left( {1 - n} \right)/2}} $$

Upon substituting Eq. (21) into (20), follow the recurrent expressions for the coefficients ${a}_{n}^{*}$:

22 $$ \begin{aligned} & a_{0}^{*} = 1;\,\,\,a_{1}^{*} = \frac{\beta }{2\sqrt \alpha };\,\,\,a_{2}^{*} = 0;\,\,\,a_{3}^{*} = - \frac{\beta }{8\sqrt \alpha };\,\,\,a_{4}^{*} = 0; \\ & a_{5}^{*} = \frac{{\ddot{\beta }}}{16\sqrt \alpha };\,\,\,a_{6}^{*} = - \frac{5}{128}\frac{{\dot{\beta }^{2} }}{\alpha };\,\,\,a_{7}^{*} = - \frac{5}{128}\frac{{\ddot{\beta }}}{\sqrt \alpha };\,\,\,a_{8}^{*} = \frac{25}{{512}}\frac{{\dot{\beta }\ddot{\beta }}}{\alpha }; \\ & a_{9}^{*} = \frac{7}{256}\frac{{\beta^{IV} }}{\sqrt \alpha } - \frac{15}{{512}}\frac{{\dot{\beta }^{3} }}{{\alpha^{3/2} }};\,\,\,\,a_{10}^{*} = - \frac{109}{{1024}}\frac{{\dot{\beta }\dddot \beta }}{\alpha } - \frac{13}{{256}}\frac{{\ddot{\beta }^{2} }}{\alpha }; \ldots \\ & \ldots \ldots \ldots \ldots \ldots \ldots \ldots \ldots \ldots \ldots \ldots \ldots \ldots \\ & \mathop {\mathop \sum \limits_{k = 0} }\limits^{m + 2k} \mathop {\mathop \sum \limits_{m = 0} }\limits^{ \le n + 1} \left( {\begin{array}{*{20}c} {\frac{1 - m}{2}} \\ k \\ \end{array} } \right)a_{m}^{*} a_{n + 1 - m - 2k}^{*} - \frac{{\beta a_{n}^{*} }}{\sqrt \alpha } = 0 \\ \end{aligned} $$

Hence, the solution is given by Eqs. (13) and (22).

To illustrate how the method works in practice, consider several particular examples.

First, consider the case $\beta =bt+d$. Assume $\alpha =1$ in the original heat equation (this is easily achieved by rescaling of the spatial variable as $x\to x\sqrt{\alpha }$).

In this case, the fractional operator given by Eq. (19) satisfies Eq. (5) with

23 $$ {\mathfrak{F}} = \frac{bt + d}{2} + \mathop {\mathop \sum \limits_{n = 0} }\limits^{\infty } c_{n} b^{n} \partial^{{\left( {1 - 3n} \right)/2}} , $$

where

24 $$ \begin{aligned} c_{0} & = 1;\,\,\,c_{1} = - \frac{1}{8};\,\,\,c_{2} = - \frac{5}{128};\,\,\,\,c_{3} = - \frac{5}{512};\,\,\,c_{4} = - \frac{1105}{{2^{15} }}; \\ c_{5} & = - \frac{1695}{{2^{15} }};\,\,\,c_{6} = - \frac{414125}{{2^{22} }};\,\,\,\,c_{7} = - \frac{59025}{{2^{18} }}; \ldots \\ 2c_{n} & = - \left( {1 - \frac{3}{4}n} \right)c_{n - 1} - \mathop {\mathop \sum \limits_{k = 1} }\limits^{n - 1} c_{k} cn - k,\,\,\,\,n \ge 1 \\ \end{aligned} $$

Hence, the solution is given by Eqs. (13), (23), and (24).

Next, consider the case, when $\beta (t)$ is a decreasing function.

If, for instance, the decreasing function $\beta (t)$ is given in the form of a series with respect to exponential functions

25 $$ \beta = \mathop {\mathop \sum \limits_{n = 0} }\limits^{\infty } \beta_{n} e^{ - nct} ,\,\,\,\,c,\beta_{n} = const, $$

then it is possible to find the operator $\mathfrak{D}$ from Eq. (5) in the form different from Eq. (6), which is the main advantage of the method leading to the form given by Eq. (19).

For the sake of illustration, consider a particular case

26 $$ \beta = be^{ - ct} ,b,c = const. $$

Substituting $\mathfrak{D}=\mathfrak{F}+\beta (t)/2$ into Eq. (5) yields the expression for $\mathfrak{F}$

27 $$ {\mathfrak{F}}^{2} + {\mathfrak{F}}\frac{\beta }{2} - \frac{\beta }{2}{\mathfrak{F}} = \partial $$

The operator $\mathfrak{F}$ is sought in the form

28 $$ {\mathfrak{F}} = \mathop {\mathop \sum \limits_{n = 0} }\limits^{\infty } b^{n} e^{ - nct} {\mathfrak{F}}_{n} , $$

where ${\mathfrak{F}}_{n}$ are operators of the same type as $\mathfrak{D}$ but with constant coefficients.

Recurrent expressions for ${\mathfrak{F}}_{n}$ are obtained from substituting Eq. (28) into (27). It happens that ${\mathfrak{F}}_{0}={\partial }^{1/2}$, while the other operators can be found in the form of convolution

29 $$ {\mathfrak{F}}_{n} f\left( t \right) = \int_{0}^{t} K_{n} \left( {t - \zeta } \right)f\left( \zeta \right)d\zeta , $$

where the kernels ${K}_{n}$ can be found by Laplace transforms in the course of the solution procedure.

For the case given by Eq. (26), it is possible to find the first two terms in the series of Eq. (28) in a simpler way—directly from Eq. (16)—after noticing that, for $c\to \infty$, it is necessary to keep only those of them, which contain ${e}^{-ct}$ (except the first one). Thus,

30 $$ \begin{aligned} - q_{s} \sqrt \alpha & \approx \left( {\partial^{1/2} + \frac{\beta }{2\sqrt \alpha } - \frac{{\mathop \beta \limits^{\cdot} }}{8\sqrt \alpha }\partial^{ - 1} + \frac{{\mathop \beta \limits^{\cdot\cdot} }}{16\sqrt \alpha }\partial^{ - 2} - \frac{5}{128}\frac{{\mathop \beta \limits^{\cdot\cdot\cdot} }}{\sqrt \alpha }\partial^{ - 3} + \cdots } \right)T_{s} \\ & = \left[ {\partial^{1/2} + \frac{b}{\sqrt \alpha }e^{ - ct} \left( {\frac{1}{2} + \frac{c}{8}\partial^{ - 1} + \frac{{c^{2} }}{16}\partial^{ - 2} + \frac{{5c^{3} }}{128}\partial^{ - 3} + \cdots } \right)} \right]T_{s} \\ \end{aligned} $$

Treating the operator $\partial$ as a constant, Eq. (30) can be written as

31 $$ - q_{s} \sqrt \alpha = \left[ {\partial^{1/2} + \frac{b}{\sqrt \alpha }e^{ - ct} \partial \left( {1 - \sqrt {1 - c\partial^{ - 1} } } \right)} \right]T_{s} $$

Upon rewriting $\sqrt{1-c{\partial }^{-1}}$ in the latter equation as $\sqrt{1-\frac{c}{\partial }}=\sqrt{\partial -c}{\partial }^{-1/2}$, the solution becomes

32 $$ - q_{s} \sqrt \alpha \approx \left[ {\partial^{1/2} + \frac{b}{\sqrt \alpha }e^{ - ct} \partial \left( {1 - e^{ct} \partial^{1/2} e^{ - ct} \partial^{ - 1/2} } \right)} \right]T_{s} $$

The equivalence between Eqs. (32) and (30) can be checked by using the product rule for fractional operators^12^, that is,

33 $$ \partial^{\nu } \left[ {f\left( t \right)g\left( t \right)} \right] = \mathop {\mathop \sum \limits_{n = 0} }\limits^{\infty } \left( {\begin{array}{*{20}c} \nu \\ n \\ \end{array} } \right)\partial^{n} f\left( t \right) \cdot \partial^{\nu - n} g\left( t \right) $$

To conclude this section, consider a very important case, when $\beta \to \varepsilon \beta (t)$, where $\varepsilon$ is a parameter. The equation for the fractional operator $\mathfrak{D}$ in Eq. (14) becomes

34 $$ {\mathfrak{D}}^{2} - \varepsilon \beta \left( t \right){\mathfrak{D}} = \partial $$

In this case, the operator $\mathfrak{D}$ can be found in the form of a power series with respect to the parameter $\varepsilon$:

35 $$ {\mathfrak{D}} = \mathop {\mathop \sum \limits_{n = 0} }\limits^{\infty } \varepsilon^{n} {\mathfrak{D}}_{n} $$

Upon substituting Eq. (35) into (34) and equating expressions at the equal powers of $\varepsilon$, follow recurrent expressions for the operators ${\mathfrak{D}}_{n}$:

36 $$ \begin{aligned} & {\mathfrak{D}}_{0}^{2} = \partial ;{\mathfrak{D}}_{0} = \partial^{1/2} \\ & {\mathfrak{D}}_{1} \partial^{1/2} + \partial^{1/2} {\mathfrak{D}}_{1} - \beta \left( t \right)\partial^{1/2} = 0 \\ & {\mathfrak{D}}_{2} \partial^{1/2} + \partial^{1/2} {\mathfrak{D}}_{2} + {\mathfrak{D}}_{1} {\mathfrak{D}}_{1} - \beta \left( t \right){\mathfrak{D}}_{1} = 0 \\ & \ldots \ldots \ldots \ldots \ldots \ldots \ldots \ldots \ldots \ldots \ldots \ldots \ldots \ldots \ldots \ldots \ldots \\ \end{aligned} $$

By direct substitution, it is easy to see that the expression for ${\mathfrak{D}}_{1}$ is given by a simple formula

37 $$ {\mathfrak{D}}_{1} = \partial^{1/2} \int_{0}^{t} \beta d\zeta \cdot \partial^{1/2} - \int_{0}^{t} \beta d\zeta \cdot \partial $$

The solution of a more general operational equation

38 $$ {\mathfrak{F}}\partial^{1/2} + \partial^{1/2} {\mathfrak{F}} - \beta \left( t \right)\mathop \sum \limits_{\nu } \partial^{\nu } = 0 $$

can be found in the same way, rendering

39 $$ {\mathfrak{F}} = \mathop \sum \limits_{\nu } \left( {\partial^{1/2} \int_{0}^{t} \beta \left( \zeta \right)d\zeta \cdot \partial^{\nu } - \int_{0}^{t} \beta \left( \zeta \right)d\zeta \cdot \partial^{\nu + 1/2} } \right) $$

The latter formula allows one to find finite expressions for all ${\mathfrak{D}}_{n}$ ($n>1$), if $\beta (t)$ is a polynomial in $t$.

## Determination of the front propagation law in Stefan-type problems at large times

It is known that problems of finding the front propagation speed can be modelled by a functional equation, which relates the dependent variable (e.g., temperature, mass concentration), its gradient at the moving boundary, and an auxiliary physical condition characterising the process under consideration^3^. For small and medium values of $t$, such a relation is given by Eqs. (16) and (19). An approximate solution of the corresponding inverse problem—determining $\beta (t)$—can be found out by using finite intervals of series in the mentioned equations.

This section is devoted to determination of the front propagation law ${\fancyscript{l}}={\int }_{0}^{t}\beta (\zeta )d\zeta$ for large values of $t$. As will be seen from the following analysis, the asymptotic equations relating ${T}_{s}$, ${q}_{s}$, and ${\fancyscript{l}}$ are quite simple. The latter provides an efficient way to determine the front propagation speed for processes, which are characterised by a nonlinear boundary condition on the front of a phase or chemical change.

Consider Eq. (16) assuming that ${T}_{s}(t)$ and ${\fancyscript{l}}(t)$ are monotonically increasing functions—${T}_{s}(t)=const$ is also possible—and ${\fancyscript{l}}(t)$ has all derivatives with respect to $t$. Assume also that, for $t\to \infty$, the surface temperature increases not faster than ${t}^{\mu }$, $0\le \mu <\infty$; the derivatives of ${T}_{s}(t)$ increase not faster than a power function as well.

Assume now that, for $t\to \infty$, ${\fancyscript{l}}(t)/\sqrt{t}\to 0$ (the front propagates slower than $\sqrt{t}$). From substituting ${\fancyscript{l}}={t}^{\nu }$ into Eq. (16) and taking into account Eq. (17), it follows that, for $t\to \infty$ and $0\le \nu <1/2$, the first term in the series dominates and, hence,

40 $$ - q_{s} \sqrt \alpha \approx \partial^{1/2} T_{s} \left( t \right),t \to \infty . $$

The same conclusion can be drawn upon assuming ${\fancyscript{l}}=\sqrt{t}/{ln}^{\varepsilon }t$, $\varepsilon >0$.

The latter equation does not contain the front propagation law and is identical with the steady case (the boundary is at rest). This can be easily understood after recalling that, within a medium at rest, heat propagates as $\sqrt{t}$; hence, if the surface temperature increases quite slow, the slowly moving front has no influence upon the heat transfer process at large $t$.

In the case when ${\fancyscript{l}}(t)/\sqrt{t}\to \infty$ for $t\to \infty$ (the front propagates faster than $\sqrt{t}$), the values of the terms in the series of Eq. (16) increase with $n$. However, one of the terms in each multipliers at each fractional derivative ${\partial }^{(1-n)/2}$ dominates. Consequently, neglecting all non-dominant terms yields

41 $$ - q_{s} \sqrt \alpha \approx \left[ {\frac{\beta }{2\sqrt \alpha } + \mathop {\mathop \sum \limits_{n = 0} }\limits^{\infty } \left( {\begin{array}{*{20}c} \frac{1}{2} \\ n \\ \end{array} } \right)\left( {\frac{{\beta^{2} }}{4\alpha }} \right)^{n} \partial^{1/2 - n} - \frac{{\mathop \beta \limits^{\cdot} }}{8\sqrt \alpha }\mathop {\mathop \sum \limits_{n = 0} }\limits^{\infty } ( - 1)^{n} \left( {\frac{{\beta^{2} }}{4\alpha }} \right)^{n} \partial^{ - 1 - n} } \right]T_{s} $$

The latter equation can be written in the finite form as

42 $$ - q_{s} \sqrt \alpha \approx \frac{\beta }{2\sqrt \alpha }T_{s} + K_{1} - K_{1} $$

where the operators ${K}_{1}$ and ${K}_{2}$ are ${K}_{1}=exp\left[{\left(-\frac{{\beta }^{2}}{4\alpha }\right)}^{*}t\right]{\partial }^{1/2}exp\left[{\left(\frac{{\beta }^{2}}{4\alpha }\right)}^{*}t\right]{T}_{s}$ and ${K}_{2}=\frac{\stackrel{\cdot }{\beta }}{8\sqrt{\alpha }}{\int }_{0}^{t}exp\left[-{\left(\frac{{\beta }^{2}}{4\alpha }\right)}^{*}(t-\zeta )\right]{T}_{s}(\zeta )d\zeta$, respectively.

The symbol $({)}^{*}$ means that, while calculating ${\partial }^{1/2}$ by Eq. (3), ${\beta }^{2}/(4\alpha )$ is to be treated as a constant independent of $t$.

Because, for $t\to \infty$, ${e}^{-ct}{\partial }^{1/2}{e}^{ct}{T}_{s}=\sqrt{\partial +c}{T}_{s}\approx {T}_{s}\sqrt{c}$ if $c=const$ and ${T}_{s}(t)$ is a power function, it follows that ${K}_{1}\to \frac{\beta }{2\sqrt{\alpha }}{T}_{s}$ and, using the mean value theorem, ${K}_{2}\to \frac{\stackrel{\cdot }{\beta }\sqrt{\alpha }}{2{\beta }^{2}}{T}_{s}.$

Keeping the dominant terms only in Eq. (42) renders

43 $$ - \alpha q_{s} \left( t \right) \approx \beta \left( t \right)T_{s} \left( t \right),t \to \infty , $$

which has the same form as the expression obtainable in the case of $\beta =const$ ^7^.

In an intermediate case, when, for instance, the boundary moves by a parabolic law ${\fancyscript{l}}(t)/\sqrt{t}\to const$ for $t\to \infty$, all terms in the series of Eq. (16) are of the same order of magnitude. In this case, the relationship between ${T}_{s}$ and ${q}_{s}$ can be written in the form similar to Eqs. (40) and (43)

44 $$ q_{s} \sqrt \alpha \approx C_{1} \partial^{1/2} T_{s} \left( t \right) \approx C_{2} \frac{\beta }{\sqrt \alpha }T_{s} \left( t \right),t \to \infty . $$

The latter expression, in contrast with Eqs. (40) and (43) contains constant coefficients ${C}_{1}$ and ${C}_{2}$, which can be found by the methods developed for the case $\beta =\stackrel{\cdot }{{\fancyscript{l}}}\sim 1/\sqrt{t}$^3^.

Now assume that, for $t\to \infty$, ${T}_{s}(t)$ increases exponentially as ${e}^{\alpha t}$ or ${t}^{\mu }{e}^{\alpha t}$, $0\le \mu <\infty$.

First, consider the general case ${\fancyscript{l}}(t)/t\to 0$ for $t\to \infty$ (the front propagates slower than $t$). Upon substituting ${\fancyscript{l}}={t}^{\nu }$ ($0\le \nu <1$) into Eq. (16) and taking into account that ${\partial }^{\nu }{t}^{\mu }{e}^{\alpha t}\to {\alpha }^{\nu }{t}^{\mu }{e}^{\alpha t}$, it follows that the first term dominates the entire series. Hence, the asymptotic solution is given by Eq. (40).

In the case when ${\fancyscript{l}}(t)/t\to \infty$ for $t\to \infty$ (the front propagates faster than $t$), analysis of Eq. (16) renders the asymptotic solution given by Eq. (43).

In the intermediate case of ${\fancyscript{l}}(t)/t\to \beta =const$ for $t\to \infty$ (the front propagates with a constant speed), the problem has the straightforward solution

45 $$ q_{s} \sqrt \alpha \approx \left[ {\frac{\beta }{2\sqrt \alpha } + exp\left( { - \frac{{\beta^{2} t}}{4\alpha }} \right)\partial^{1/2} exp\left( {\frac{{\beta^{2} t}}{4\alpha }} \right)} \right]T_{s} ,t \to \infty . $$

The latter expression can be written in one of the forms given by Eq. (44), too.

Thus, the asymptotic expressions for the front propagation speed, for which the law relating ${T}_{s}$, ${q}_{s}$ and $\beta$ changes its form, have been obtained. The regimes change for various values of $\nu$ depending on how the surface temperature ${T}_{s}(t)$ increases.

To conclude this section, consider an illustrative example ${T}_{s}=At$ with $A=const$ and the auxiliary condition on the boundary in the form

46 $$ - \alpha q_{s} = Q\beta^{k} , $$

where $Q>0$ and $k>0$ are both constants.

It is necessary to determine the front propagation law for $t\to \infty$.

Consider two assumptions, ${\fancyscript{l}}/\sqrt{t}\to 0$ and ${\fancyscript{l}}/\sqrt{t}\to \infty$.

In the first case, combining Eqs. (46) and (40) yields

47 $$ \ell \approx \left( {\frac{2A}{Q}\sqrt {\frac{\alpha }{\pi }} } \right)^{1/k} \frac{2k}{{2k + 1}}t^{{1 + 1/\left( {2k} \right)}} $$

In the second case, from Eqs. (46) and (43) follows

48 $$ \ell \approx \left( \frac{A}{Q} \right)^{{1/\left( {k - 1} \right)}} \frac{k - 1}{k}t^{{k/\left( {k - 1} \right)}} $$

Equation (47) cannot be valid, because from this equation it follows that, for $t\to \infty$, the front propagates faster than $\sqrt{t}$. The latter contradicts the initial assumption. Equation (48) renders the solution of the problem.

In principle, one can arrive at the results similar to Eqs. (47, 48) by a laborious heat-balance integral method^20^. However, the latter requires solving multiple intermediate and auxiliary differential equations. And, which is even more important, the knowledge of the entire temperature field is needed. On the contrary, the way, proposed here, yields an identical result from a single formula. In this lies a great advantage of the method employed in this study.

It is worth noting, however, that the regimes logarithmically close to the critical ones, for instance, ${\fancyscript{l}}\sim \sqrt{t}{ln}^{\varepsilon }t$ or ${\fancyscript{l}}\sim t{ln}^{\varepsilon }t$ for ${T}_{s}\sim {t}^{\mu }$ and ${T}_{s}\sim {t}^{\mu }{e}^{\alpha t}$, respectively, are not yet studied.

## General discussion

The non-field solution to the problem of heat transfer through a boundary moving with a constant speed has been obtained by the method of Kulish earlier^14^. In a later study, the behaviour of this solution has been investigated to establish a set of conditions, under which chaotic solutions become possible^15^. However, the case of the boundary moving arbitrarily remained outside the study, mostly due to the fact that the series form of the fractional operator was not known until recently^18^.

Some attempts to apply the methods of fractional calculus to problems with phase change on the boundary^21^ and combustion^22^ have also been made. But again, the cases, considered in those studies, are rather special and lack the generality presented in this work. In particular, the solutions, reported in the mentioned works, are approximate, because, to find them, non-compact fractional operators with slow convergence have been used.

The extension of the method of Kulish, presented in this work, allows not only to establish a compact form of the fractional operator, which ensures much faster convergence of results, but also obtain non-field solutions, which relate local values of the temperature and its gradient, in the case of the boundary moving arbitrarily. In most of the cases, these solutions either cannot be obtained by other analytical methods, or the procedures leading to these solutions are too laborious (e.g., involve a large volume of calculations), which makes them impractical.

Among the cases investigated in this study are such cases as the boundary speed changing linearly, parabolically, exponentially, and polynomially. Whenever possible, the solutions thus obtained have been compared with known solutions. In addition, section “Determination of the front propagation law in Stefan-type problems at large times” is fully devoted to determination of the front propagation law in Stefan-type problems at large times.

Last but not least, it has been shown that, for Stefan-type problems, where applications of the non-field method are practically impossible due to a large volume of necessary computations, it is still possible to analyse the solution behaviour at large times and, in many cases, find asymptotic solutions. Obviously, nowadays all necessary computations can be fulfilled by built-in algorithms on public software (e.g., Matlab, Maple, Mathematica, Derive, etc.); however, in many practical engineering applications, the knowledge gained from asymptotic analysis is sufficient to provide desired estimates.

## List of symbols

${a}_{n}, {a}_{n}^{*}, {c}_{n}$: Coefficients in fractional series
$$b, c, d$$: Arbitrary constants
$$C, {C}_{1}, {C}_{2}$$: Arbitrary constants
*f*, *g*: Arbitrary functions of independent variables
*k*, *n*, *m*: Summation indices
$${K}_{n}, {K}_{1}, {K}_{2}$$: Kernels
$p$: Laplace transform variable
*q*: Temperature gradient
$$Q$$: Auxiliary constant
*T*: Temperature
*t*: Time
*x*: Spatial variable

## Greek

*α, β, γ*$$, \delta $$: Arbitrary functions
Θ: Temperature jump
$$\varepsilon $$: Parameter
$$\zeta $$: Dummy variable
$\mu ,$*ν*: Order of fractional differ-integration

## Special symbols

${\partial }^{\nu }$: Fractional differential operator of order *ν*
$$\mathfrak{D}, \mathfrak{F}$$: Fractional operators
$\mathfrak{L}$: Laplace transform operator
${\fancyscript{l}}$: Front propagation law

## References

[1] Novozhilov BV (1970). Equation for non steady-state combustion velocity of a powder. J. Appl. Mech. Tech. Phys..

[2] Tikhonov, A. N. & Samarskii, A. A. *Equations of Mathematical Physics* (Pergamon Press, 1963).

[3] Hahn DW, Özisik MN (2012). Heat Conduction.

[4] Rubinstein L (1971). The Stefan Problem.

[5] Pogorzelski W (1966). Integral Equations and Their Applications.

[6] Turkyilmazoglu M (2016). Heat transfer from warm water to a moving foot in a footbath. Appl. Therm. Eng..

[7] Turkyilmazoglu M (2017). Cooling of particulate solids and fluid in a moving bed heat exchanger. J. Heat Transf..

[8] Turkyilmazoglu M (2018). Stefan problems for moving phase change materials and multiple solutions. Int. J. Thermal Sci..

[9] Turkyilmazoglu M (2020). Combustion of a solid fuel material at motion. Energy.

[10] Myers TG, Hennessy MG, Calvo-Schwarzwälder M (2020). The Stefan problem with variable thermophysical properties and phase change temperature. Int. J. Heat Mass Transf..

[11] Frankel JI (2006). Generalising the method of Kulish to one-dimensional unsteady heat conducting slabs. J. Thermophys. Heat Transf..

[12] Kulish VV, Lage LJ (2000). Fractional-diffusion solutions for transient local temperature and heat flux. J. Heat Transf..

[13] Kulish VV, Lage JL (2002). Application of fractional calculus to fluid mechanics. J. Fluids Eng..

[14] Poletkin K, Kulish V (2012). A generalised relation between the local values of temperature and the corresponding heat flux in a one-dimensional semi-infinite domain with the moving boundary. Int. J. Heat Mass Transf..

[15] Kulish, V. V., Horák, V., Linh Do Duc. A generalised relation between the local values of temperature and the corresponding heat flux in a one-dimensional semi-infinite domain with the moving boundary: Investigation of behaviour. *AIP Proc.*10.1063/1.5081576 (2018).

[16] Kulish VV, Heng Li, Dröge P (2007). Z-DNA-induced super-transport of energy within genomes. Phys. A..

[17] Kulish VV (2008). Market efficiency and the phase-lagging model of price fluctuations. Phys. A.

[18] Kulish V (2020). A non-field analytical method for solving energy transport equations. J. Heat Transf..

[19] Oldham KB, Spanier J (1974). The Fractional Calculus.

[20] Font F, Mitchell SL, Myers TG (2013). One-dimensional solidification of supercooled melts. Int. J. Heat Mass Transf..

[21] Kulish, V. V., Horák, V., Linh D. D. & Tomáš L. On the possibility to develop an advanced non-equilibrium model of depressurisation in two-phase fluids. *AIP Proc.*10.1063/1.4972639 (2017).

[22] Kulish, V. V., Horák, V., Linh D. D. & Tomáš, L. Application of fractional calculus to modelling transient combustion of solid propellants. *AIP Proc.*10.1063/1.4972680 (2017).

